# The evolution of neuropeptide signalling: insights from echinoderms

**DOI:** 10.1093/bfgp/elx005

**Published:** 2017-04-21

**Authors:** Dean C. Semmens, Maurice R. Elphick

**Keywords:** neuropeptide, evolution, genomics, echinoderms, sea urchin, starfish

## Abstract

Neuropeptides are evolutionarily ancient mediators of neuronal signalling that regulate a wide range of physiological processes and behaviours in animals. Neuropeptide signalling has been investigated extensively in vertebrates and protostomian invertebrates, which include the ecdysozoans *Drosophila melanogaster* (Phylum Arthropoda) and *Caenorhabditis elegans* (Phylum Nematoda). However, until recently, an understanding of evolutionary relationships between neuropeptide signalling systems in vertebrates and protostomes has been impaired by a lack of genome/transcriptome sequence data from non-ecdysozoan invertebrates. The echinoderms—a deuterostomian phylum that includes sea urchins, sea cucumbers and starfish—have been particularly important in providing new insights into neuropeptide evolution. Sequencing of the genome of the sea urchin *Strongylocentrotus purpuratus* (Class Echinoidea) enabled discovery of (i) the first invertebrate thyrotropin-releasing hormone-type precursor, (ii) the first deuterostomian pedal peptide/orcokinin-type precursors and (iii) NG peptides—the ‘missing link’ between neuropeptide S in tetrapod vertebrates and crustacean cardioactive peptide in protostomes. More recently, sequencing of the neural transcriptome of the starfish *Asterias rubens* (Class Asteroidea) enabled identification of 40 neuropeptide precursors, including the first kisspeptin and melanin-concentrating hormone-type precursors to be identified outside of the chordates. Furthermore, the characterization of a corazonin-type neuropeptide signalling system in *A. rubens* has provided important new insights into the evolution of gonadotropin-releasing hormone-related neuropeptides. Looking forward, the discovery of multiple neuropeptide signalling systems in echinoderms provides opportunities to investigate how these systems are used to regulate physiological and behavioural processes in the unique context of a decentralized, pentaradial bauplan.

## Neuropeptide signalling systems: evolutionarily ancient regulators of physiology and behaviour

Neuropeptides are intercellular signalling molecules that are secreted by neurons to act as neurotransmitters, neuromodulators or neurohormones [1]. They are the largest and most diverse class of signalling molecules in the nervous system [2], ranging in size from just three amino acids [e.g. thyrotropin-releasing hormone (TRH)] [3] to much longer polypeptides [e.g. corticotropin-releasing hormone (CRH), which comprises 41 residues] [4]. However, all neuropeptides share the common characteristic of being derived from larger precursor proteins, which have an N-terminal signal peptide that targets the precursor protein to the regulated secretory pathway [5]. In addition, precursor proteins have canonical cleavage sites (e.g. monobasic and/or dibasic sites recognized by prohormone convertases [6]) and sites for post-translational modification (e.g. a C-terminal glycine residue is often a substrate for amidation, which can be crucial for bioactivity [7]). Neuropeptides, with a few exceptions, typically bind to and activate G-protein coupled receptors (GPCRs) belonging to the rhodopsin-β, rhodopsin-γ and secretin-type receptor families [8].

The evolutionary origins of neuropeptides as regulators of physiology and behaviour are ancient and a number of neuropeptide signalling systems have been traced back to the common ancestor of bilaterian animals (Urbilateria) >550 million years ago [9, 10]. Furthermore, neuropeptide signalling pathways are also key components of the nervous systems in sister phyla to the bilaterians (e.g. cnidarians [11]), and the origins of some peptide signalling pathways may pre-date the emergence of animals with nervous systems [12].

Historically, establishing relationships between neuropeptide signalling systems in evolutionarily distant phyla was possible for neuropeptides with highly conserved structures. For example, vasopressin/oxytocin (VP/OT)-type peptides comprise a characteristic disulphide bridge between cysteine residues at positions 1 and 6 of the mature peptide that is crucial for bioactivity and which is conserved in members of this neuropeptide family throughout the Bilateria [13, 14]. Furthermore, it has been found that VP/OT-type peptides regulate reproductive behaviour in both vertebrates and invertebrates, providing evidence of evolutionary conservation of not only neuropeptide structure but also neuropeptide function [15]. However, perhaps more typically, there is relatively little sequence similarity shared by related neuropeptides from different phyla and therefore establishing relationships is difficult when only the primary amino acid sequence of bioactive neuropeptides is known. Nevertheless, in the pre-genomic era, evidence of the evolutionarily ancient origins of neuropeptide signalling systems was obtained based on primary sequence similarity [16], cross-immunoreactivity [17] or functional similarity [18].

## Neuropeptide relationships: insights from the first animal genome sequences

The turn of the 21st century heralded the beginning of the post-genomic era, and sequencing of the genomes of the nematode *Caenorhabditis elegans* in 1998 [19], the fruit-fly *Drosophila melanogaster* in 2000 [20] and *Homo sapiens* in 2001 [21] enabled the first comprehensive analyses of genes encoding neuropeptide precursors and receptors in these species [8, 22, 23]. Subsequently, deorphanization of candidate neuropeptide receptors provided important new insights into the evolutionary relationships and functional diversity of neuropeptide signalling systems [24–30]. Furthermore, in some cases neuropeptide receptor deorphanization revealed unexpected relationships. This is perhaps best exemplified by the unification of gonadotropin-releasing hormone (GnRH) and adipokinetic hormone (AKH) as members of the same neuropeptide family.

Insect AKHs are lipid-mobilizing hormones released during flight and locomotion [31]. In 2002, the receptor for *Drosophila* AKH (pQLTFSPDWG-NH_2_) was identified and pharmacologically characterized [32, 33]. Interestingly, it was found that insect AKH receptors are structurally and evolutionarily related to vertebrate GnRH receptors. In mammals, GnRH controls reproductive maturation and function by stimulating release of luteinizing hormone and follicle-stimulating hormone from the pituitary gland [34, 35], but mammalian GnRH (e.g. human GnRH is pQHWSYGLRPG-NH_2_) shares only modest sequence similarity with AKH. Thus, the discovery of insect AKH receptors enabled unification of a bilaterian neuropeptide family that hitherto had not been recognized based on primary sequence similarity or biological activity.

## Neuropeptide evolution: insights from the genome sequences of species from an increasing variety of animal phyla

Recently, genome sequence data have been obtained from an increasing variety of phyla, expanding the scope for genome-wide investigation of neuropeptide signalling systems beyond the vertebrates and ‘model’ invertebrates such as *D. melanogaster* and *C. elegans*, which are both ecdysozoan protostomian invertebrates. For example, analysis of the repertoire of GPCRs in invertebrate chordates—the urochordate *Ciona intestinalis* [36] and the cephalochordate *Branchiostoma floridae* [37]—has revealed both loss and expansion of some neuropeptide receptor families. For example, in *B. floridae* there appears to have been an expansion of rhodopsin-type receptors related to mammalian neuropeptide FF (NPFF) receptors [37].

The availability of genome sequence data has also enabled genome-wide investigation of neuropeptide signalling systems in lophotrochozoan protostomes, including the mollusc *Lottia gigantea* [38] and the annelids *Capitella teleta* and *Helobdella robusta* [39]. In 2010, a survey of the genome of the owl limpet *L. gigantea* identified >40 neuropeptide precursors [38]. Among these were the first homologues of bursicon, proctolin and allatostatin C to be identified in a molluscan species [38]. Subsequent surveys of the genomes of the polychaete worm *C. teleta* and the leech *H. robusta* identified 43 neuropeptide precursors in *C. teleta* and 35 neuropeptide precursors in *H. robusta* [39]. Interestingly, there were distinct differences between these two species. For example, *H. robusta* appears to have lost the bursicon-type and glycoprotein hormone-type precursors and receptors that are present in *C. teleta* [39].

In 2013, two independent studies set out to analyze the growing body of genome sequence data from a range of phyla to investigate neuropeptide relationships and neuropeptide evolution in the animal kingdom. A core set of neuropeptide-receptor signalling pathways were traced back to the common ancestor of the Bilateria [9, 10], revealing relationships between neuropeptides in protostomes and deuterostomes that were not readily apparent from comparisons of the primary amino acid sequences of known bioactive or putative neuropeptides. For example, relationships were discovered between (i) deuterostomian orexin and protostomian allatotropin; (ii) deuterostomian neuropeptide S (NPS) and protostomian crustacean cardioactive peptide (CCAP); (iii) deuterostomian NPFF and protostomian SIFamide; (iv) vertebrate gastrin-releasing peptide and endothelin and protostomian CCHamide; and (v) deuterostomian galanin and protostomian allatostatin A [10].

Of particular importance in these studies were the analysis of genome sequence data from lophotrochozoan protostomes (annelids and molluscs) and non-chordate deuterostomes (the Ambulacraria; hemichordates and echinoderms). A good example of the importance of the use of lophotrochozoan and ambulacrarian genome sequence data was the unification of a bilaterian neuropeptide family that includes allatotropin and orexin-type precursors. The allatotropins were first identified as peptides stimulating the synthesis and secretion of juvenile hormone from the *corpora allata* in insects [40, 41]. The orexins were first identified as hypothalamic neuropeptides that stimulate food intake in mammals [42, 43], but it has subsequently been discovered that orexins also stimulate wakefulness and energy expenditure [44]. The homology of allatotropins and orexins was not evident based solely on their primary amino acid sequences. However, analysis of the genome of the hemichordate *Saccoglossus kowalevskii* identified an orexin-type precursor with a conserved domain outside of the putative neuropeptide region [10]. This ‘cryptic’ domain is present in all protostomian allatotropin-type precursors but had not previously been identified in orexin-type precursors because this domain appears to have been lost in the chordates. Therefore, the analysis of genome sequence data from an ambulacrarian was crucial in unifying a bilaterian neuropeptide family.

## The echinoderms: ‘bridging the gap’ for reconstruction of neuropeptide evolution

The echinoderms are a phylum of marine organisms that together with the hemichordates form the Ambulacraria. The echinoderms comprise five extant classes—echinoids (e.g. sea urchins), holothurians (e.g. sea cucumbers), asteroids (e.g. starfish), ophiuroids (e.g. brittle stars) and crinoids (e.g. sea lilies/feather stars). The echinoids and holothurians form the echinozoan clade; the asteroids and ophiuroids form the asterozoan clade, while the crinoids are basal to the echinozoan and asterozoan clades [45, 46].

The echinoderms are particularly interesting for comparative and evolutionary studies on neuropeptide signalling systems for a number of reasons. The echinoderms are deuterostomian invertebrates and therefore ‘bridge’ a huge evolutionary gap between the chordates and model protostomian invertebrates (e.g. *D. melanogaster* and *C. elegans*), providing key insights into the evolution of neuropeptide systems in the animal kingdom. Furthermore, the echinoderms offer a unique context to investigate the evolution and diversity of neuropeptide function. The echinoderms exhibit pentaradial symmetry as adult animals that is derived from a bilateral body plan both evolutionarily and developmentally and consequently they have a decentralized nervous system [47, 48]. In addition, there is evidence that neuropeptides may be involved in mediating neural control of several unusual biological phenomena in the echinoderms including the ability to autotomize and then regenerate body parts [49] and the mutability of their collagenous tissue, which can rapidly change between stiff and soft mechanical states under the control of the nervous system [50, 51].

## The sea urchin genome yields new insights into neuropeptide evolution and diversity

The first extensive analysis of neuropeptide signalling systems in an echinoderm species was enabled by sequencing of the genome of the sea urchin *Strongylocentrotus purpuratus* (Class Echinoidea) [52]. Approximately 23 300 genes were identified in *S. purpuratus*, with representatives of nearly all vertebrate gene families [52]. The sea urchin has long been used as a model system for developmental and systems biology [53] but sequencing of the genome allowed exploration of numerous regulatory networks including the defensome, adhesome and the nervous system [52].

An initial analysis of *S. purpuratus* genome sequence data led to the identification of only a few neuropeptide precursors but 37 candidate neuropeptide receptors [48, 52]. However, subsequent analysis of 2026 expressed sequence tags from an *S. purpuratus* radial nerve cDNA library led to the identification of 20 candidate neuropeptide/peptide hormone precursors in this species [54]. These included homologues of VP/OT, GnRH, calcitonin and a number of putative neuropeptides that were not recognized as homologues of known neuropeptides [54]. Below we highlight some of the more important and interesting discoveries that emerged from analysis of neuropeptide systems in the sea urchin.

### The first TRH-type precursor to be discovered in an invertebrate

TRH was discovered as a hypothalamic peptide that stimulates the release of thyroid-stimulating hormone (TSH) and prolactin from the anterior pituitary gland in mammals. TSH then triggers the release of the thyroid hormones triiodothyronine and thyroxine that stimulate metabolism and thus promote growth and development [55]. However, in mammals, TRH also acts as a neurotransmitter or neuromodulator in other regions of the brain [56, 57]. Interestingly, in non-mammalian vertebrates (e.g. amphibians and fish), TRH stimulates the release of pituitary growth hormone and prolactin but has little or no effect on the secretion of TSH [58].

**Figure 1.**
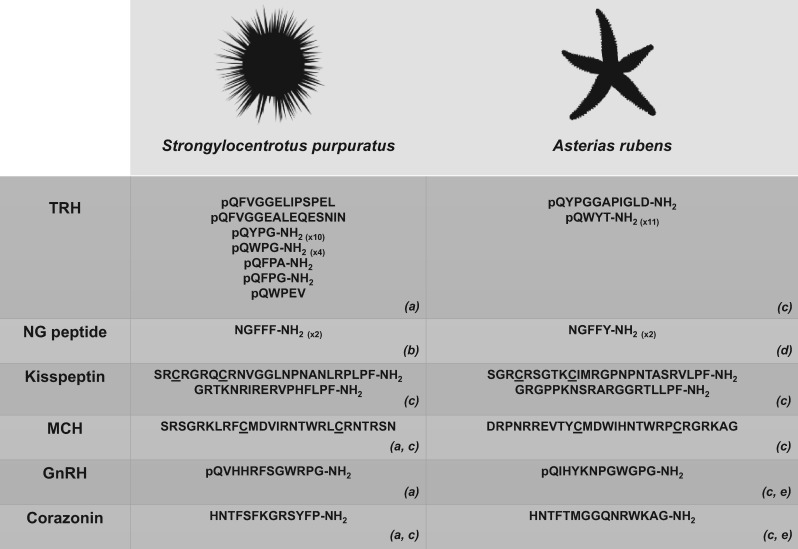
Echinoderm neuropeptides that have provided new insights into the evolution of neuropeptide signalling systems. The sequences of sea urchin (*S. purpuratus*) and starfish (*A. rubens*) representatives of six selected neuropeptide types are shown. Predicted or confirmed post-translational modifications, including conversion of an N-terminal glutamine (Q) to a pyro-glutaminyl (pQ) residue and conversion of a C-terminal glycine (G) to an amide group (-NH_2_), are depicted and cysteine (C) residues that form or are predicted to form a disulphide bridge are underlined. Numbers in parentheses represent the number of copies of the neuropeptide in the corresponding precursor if this is greater than one. The image of *S. purpuratus* was obtained from https://openclipart.org/detail/170807/sea-urchin-silhouette, while the image of *A. rubens* was created by M. Zandawala (Stockholm University). Key: TRH: thyrotropin-releasing hormone; MCH: melanin-concentrating hormone; GnRH: gonadotropin-releasing hormone. References: (a) [54]; (b) [59]; (c) [60]; (d) [61]; (e) [62].

Analysis of *S. purpuratus* radial nerve cDNA sequence data enabled discovery of the first TRH-type precursor to be identified in an invertebrate [54]. This discovery indicated that the origin of the TRH-type neuropeptide signalling system dates back at least as far as the common ancestor of deuterostomes. The *S. purpuratus* TRH-type precursor is a 316-residue precursor protein comprising a predicted 15-residue N-terminal signal peptide and 19 putative TRH-type peptides (Figure 1). These include 10 copies of the sequence QYPGG, four copies of the sequence QWPGG and single copies of the sequences QFPAG, QFPGG, QFVGGELIPSPEL, QWPEV and QFVGGEALEQESNIN [54]. These putative neuropeptides are predicted to be subject to post-translational modifications including the conversion of an N-terminal glutamine residue to a pyroglutamate and use of the C-terminal glycine as a substrate for amidation, which although not unique to TRH are nevertheless two characteristic features of vertebrate TRH-type peptides [54].

Despite the occurrence of TRH-type receptors in the protostomes [9, 10], the discovery of a TRH-type precursor in a protostomian species had, until recently, remained elusive. In 2015, it was discovered that FSEFLGamide is the ligand for a TRH-type receptor in the annelid *Platynereis dumerilii* [63]. It has therefore been proposed that the ‘EFLGamides’ identified in the lophotrochozoans [64] are orthologous to deuterostomian TRH-type peptides [63]. Thus, the evolutionary origin of the TRH-type neuropeptide signalling system dates back to the common ancestor of the Bilateria, and the discovery of the TRH-type precursor in the sea urchin *S. purpuratus* was a crucial step in providing evolutionary insights into this ancient neuropeptide family.

### The first pedal peptide/orcokinin-type neuropeptides to be discovered in deuterostomes

Pedal peptide (PLDSVYGTHGMSGFA) was first isolated from the mollusc *Aplysia californica* as a peptide that causes contraction of pedal muscles [65, 66]. In 2006, the *A. californica* pedal peptide precursor was identified through analysis of transcriptome sequence data, revealing that the precursor contains 17 copies of pedal peptide as well as two other structurally related peptides [67]. Furthermore, in *Aplysia*, there are three additional precursors containing peptides related to pedal peptide [67]. Subsequently, pedal peptide-type precursors have also been identified in other molluscan species (e.g. *L. gigantea* [38]) and in annelids (e.g. *P. dumerilii* [64] and *C. teleta* [39]).

Analysis of *S. purpuratus* radial nerve cDNA sequence data led to the discovery of the first pedal peptide-type precursors to be identified in a deuterostomian invertebrate [54]. This discovery indicated that the origins of pedal peptide-type signalling dates back to the common ancestor of the Bilateria. The *S. purpuratus* pedal peptide-type precursor 1 (SpPPLNP1) is a 510-residue protein comprising a 29-residue N-terminal signal peptide and 21 copies of pedal peptide-like peptides (SpPPLN1a-i). The *S. purpuratus* pedal peptide-type precursor 2 (SpPPLNP2) is a 204-residue protein comprising a 19-residue N-terminal signal peptide and 10 putative pedal peptide-like peptides (SpPPLN2a-i). Putative pedal peptides derived from both SpPPLNP1 (e.g. SpPPLN1d) and SpPPLNP2 (e.g. SpPPLN2h) share a C-terminal SGFx motif (where x is a hydrophobic residue) with pedal peptide in *Aplysia*, while also sharing similar characteristics with respect to the number of residues and distribution of hydrophobic and hydrophilic residues [54].

The discovery of SpPPLNP1 and SpPPLNP2 also enabled the identification of pedal peptide-type precursors in the nematode *C. elegans* [54] that share sequence similarity with arthropod orcokinin-type peptides [54]. Orcokinin was first isolated from abdominal nerve cord extracts of the crayfish *Orconectus limosus* on account of its effect in stimulating hindgut myoactivity [68]. Subsequently, orcokinin-type peptides have been identified in a number of arthropods and attributed a range of functions (e.g. regulation of ecdysteroidogenesis in the silk moth *Bombyx mori* [69]). The discovery of the *S. purpuratus* pedal peptide-type precursors provided a crucial step in unifying lophotrochozoan pedal peptides with ecdysozoan orcokinin-type peptides and in demonstrating the existence of a bilaterian family of pedal peptide/orcokinin-type peptides.

### NG peptides unify a bilaterian neuropeptide family

A 266-residue protein in the sea urchin *S. purpuratus* comprising a predicted 26-residue N-terminal signal peptide and two tandem copies of the sequence NGFFFG bounded by dibasic cleavage sites [59] was discovered on account of sequence similarity that its constituent neuropeptide (NGFFFamide) shares with NGIWYamide—a myoactive neuropeptide that is a potent inducer of oocyte maturation and spawning in the sea cucumber *Apostichopus japonicus* [70, 71]. A surprising feature of the NGFFFamide precursor was the presence of a C-terminal neurophysin domain [59]. Hitherto, neurophysins were thought to be a unique feature of VP/OT-type precursors, in which they are required for axonal transport and secretion of the neurohypophyseal hormones VP and OT [72].

The discovery of the sea urchin NGFFFamide precursor led to the discovery of the ‘NG peptide’ family in deuterostomian invertebrates, so called because they have in a common an asparagine (N)–glycine (G) motif [73]. Interestingly, an NG peptide precursor in the cephalochordate *B. floridae* comprises two copies of a putative neuropeptide with the sequence SFRNGVamide [73], which is identical to the N-terminal region of NPS (SFRNGVGTGMKKTSFQRAKS) in humans [74]. NPS-type peptides are found in the tetrapod vertebrates and have been shown to have anxiolytic-like effects in humans and rodents [74–76]. Furthermore, NPS has been identified as the ligand for the human receptor GPR154, which is paralogous to VP/OT-type receptors [77].

A broader phylogenetic analysis revealed that orthologues of NPS-type receptors are also found in invertebrates [9, 10]. Furthermore, the ligand that activates the NPS-type receptor in *Drosophila* is CCAP (PFCNAFTGCamide) [33], a neuropeptide that controls ecdysis behaviour in arthropods [78, 79]. NPS and CCAP share little sequence similarity and therefore the discovery that their receptors are orthologous was unexpected. However, it was noted that CCAP shares superficial sequence similarity with VP/OT-type peptides by virtue of a disulphide bridge between two cysteine residues [80]. In addition, the finding that NPS/CCAP-type receptors are paralogous to VP/OT-type receptors suggested that CCAP and VP/OT-type peptides may have evolved from a common ancestral molecule [10]. However, the relationship between NPS and VP/OT-type peptides or CCAP was unclear. In this respect, the discovery of NG peptides in echinoderms and other deuterostomian invertebrates was crucial in providing the ‘missing link’ between previously unassociated neuropeptide signalling systems.

**Figure 2.**
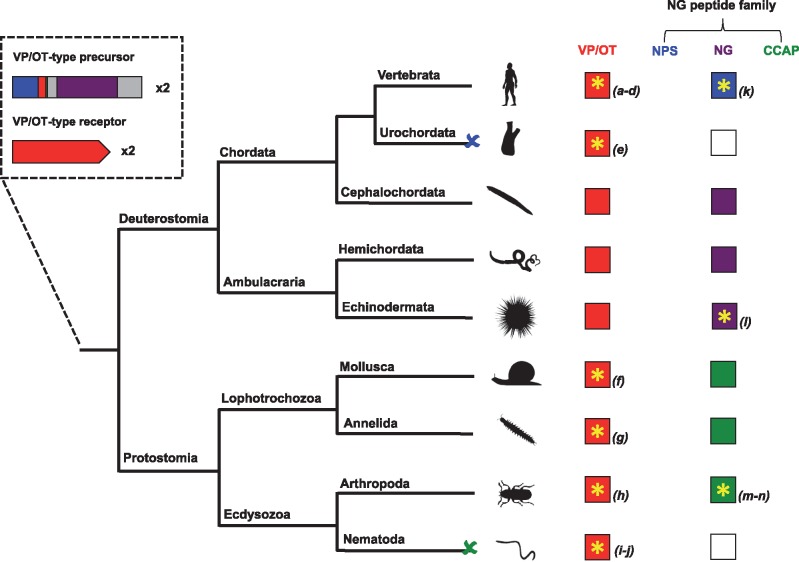
Evolution of the VP/OT-type and NG peptide signalling systems. The diagram shows how duplication of a VP/OT-type neuropeptide signalling system in the common ancestor of the Bilateria gave rise to the highly conserved VP/OT-type (red boxes) and the divergent NPS (blue boxes), NG peptide (purple boxes) and CCAP-type (green boxes) signalling systems in extant bilaterians. Phyla where neuropeptide ligand–receptor pairs have been pharmacologically characterized are labelled with a yellow asterisk. A blue cross (and white box) represents loss of the NPS-type signalling system in the urochordates, while a red cross (and white box) represents loss of the CCAP-type signalling system in the nematodes. The image of *S. purpuratus* was obtained from https://openclipart.org/detail/170807/sea-urchin-silhouette, while images of other representative species from each phylum were obtained from http://phylopic.org or were created by the authors or by M. Zandawala (Stockholm University). References: (a) [84]; (b) [85]; (c) [86]; (d) [87]; (e) [88]; (f) [89]; (g) [63]; (h) [90]; (i) [91]; (j) [92]; (k) [74]; (l) [82]; (m) [93]; (n) [94]. (A colour version of this figure is available online at: https://academic.oup.com/bfg)

Analysis of genome sequence data revealed that NPS/CCAP-type receptors are also present in deuterostomian invertebrates including in the sea urchin *S. purpuratus* [10, 81]. In accordance with sequence similarity shared by SFRNGVamide in the cephalochordate *B. floridae* and NPS (SFRNGVGTGMKKTSFQRAKS) in tetrapod vertebrates, it was hypothesized that the NG peptides may be the ligands for the NPS/CCAP-type receptors in deuterostomian invertebrates. Crucially, it has recently been shown that the NG peptide NGFFFamide is present in extracts of the sea urchin *S. purpuratus* and activates a *S. purpuratus* NPS/CCAP-type receptor [82]. This finding unites a bilaterian family of neuropeptides that includes NPS-type peptides in tetrapod vertebrates, NG peptides in deuterostomian invertebrates and CCAP-type peptides in protostomian invertebrates. Furthermore, it provides support for a scenario of neuropeptide-receptor evolution that has been postulated based on phylogenetic reconstruction of bilaterian neuropeptide signalling systems [10, 83]. In this evolutionary scenario, an ancestral VP/OT-type precursor gene duplicated and one copy retained the highly conserved features of VP/OT-type precursors. The second copy diverged through evolution to give rise to genes encoding NPS-type peptides in vertebrates, NG peptides in deuterostomian invertebrates and CCAP-type peptides in protostomian invertebrates (Figure 2).

**Figure 3.**
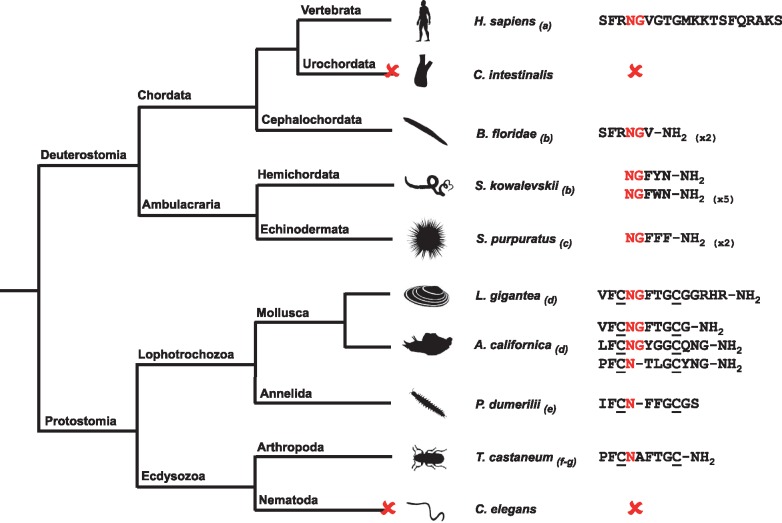
The NG peptide family. Schematic showing an alignment of putative or confirmed neuropeptide(s) derived from NPS, NG peptide and CCAP-type precursors in representative species from phyla across the Bilateria. The conserved NG motif of NPS, NG peptides and CCAP-type peptides are highlighted in red and cysteine (C) residues that form or are predicted to form a disulphide bridge are underlined. A red cross represents loss of the NPS-type signalling system in the urochordates (e.g. *C. intestinalis*) or CCAP-type signalling system in the nematodes (e.g. *C. elegans*). Numbers in parentheses represent the number of copies of the neuropeptide in the precursor if this is greater than one. The image of *S. purpuratus* was obtained from https://openclipart.org/detail/170807/sea-urchin-silhouette, while images of other representative species from each phylum were obtained from http://phylopic.org or were created by the authors or by M. Zandawala (Stockholm University). Key: *H. sapiens: Homo sapiens; C. intestinalis: Ciona intestinalis; B. floridae: Branchiostoma floridae; S. kowalevskii: Saccoglossus kowalevskii; S. purpuratus: Strongylocentrotus purpuratus; L. gigantea: Lottia gigantea; A. californica: Aplysia californica; P. dumerilii: Platynereis dumerilii; T. castaneum: Tribolium castaneum; C. elegans: Caenorhabditis elegans.* References: (a) [74]; (b) [73]; (c) [59]; (d) [38]; (e) [64]; (f) [95]; (g) [96]. (A colour version of this figure is available online at: https://academic.oup.com/bfg)

We propose that the bilaterian neuropeptide family comprising NPS/CCAP-type peptides are collectively known as NG peptides. In support of this proposal, the NG motif is not only a feature of NPS and NG peptides in deuterostomes but also a feature of CCAP-type peptides in molluscs. For example, the NG motif is present in CCAP-type peptides in the owl limpet *L. gigantea* [38] and in other molluscan species, including *Conus villepinii* (GI: 325529921) and *A. californica* (GI: 524893759) [82]. Thus, it appears that the NG motif is a unifying characteristic of this bilaterian family of neuropeptides, but with subsequent loss or substitution of the glycine residue in some CCAP-type peptides (Figure 3).

## Starfish neural transcriptome provides new insights into neuropeptide evolution and diversity

As highlighted above, analysis of the genome/transcriptome of the sea urchin *S. purpuratus* (Class Echinoidea) has demonstrated the importance of echinoderms in providing key insights into neuropeptide evolution. Analysis of transcriptome sequence data for neuropeptide-related transcripts has subsequently been extended to species belonging to other echinoderm classes. For example, analysis of the transcriptome of the sea cucumber *A. japonicus* (Class Holothuroidea) resulted in the identification of 17 neuropeptide/neurohormone precursors [97]. More recently, transcriptome sequence data obtained for the brittle star *Ophionotus victoriae* (Class Ophiuroidea) and the feather star *Antedon mediterranea* (Class Crinoidea) [98] have enabled identification of SALMFamide precursors in these species, providing new insights into the evolution of the SALMFamide family of neuropeptides in echinoderms [98].

The most extensive analysis of echinoderm neuropeptide signalling systems to date has been enabled by sequencing of the radial nerve cord transcriptome from the common European starfish *Asterias rubens* (Class Asteroidea) [60]. This led to the identification of 40 neuropeptide precursors including the first tachykinin, somatostatin, pigment-dispersing factor and CRH-type precursors to be discovered in the echinoderm/ambulacrarian clade of the animal kingdom [60]. Among the most interesting findings from this analysis, which are highlighted below, were the discovery of the first kisspeptin and melanin-concentrating hormone (MCH)-type precursors to be identified outside of the chordates [60]. Furthermore, identification of the precursors of two GnRH-like peptides in *A. rubens* provided a basis for functional characterization of receptors for these neuropeptides, which has provided new insights into the evolution of GnRH-related neuropeptide signalling systems, as also discussed below.

### The first kisspeptins to be discovered in a non-chordate

Kisspeptins are a family of structurally related neuropeptides derived from differential proteolytic processing of a precursor protein encoded by the KiSS-1 gene. The most abundant is kisspeptin-54, which can be cleaved to 14, 13 and 10 residue kisspeptins that share a common C-terminal RFamide motif [99]. Kisspeptins regulate reproductive maturation in humans and other mammals [100] by triggering the hypothalamic secretion of GnRH, which stimulates the release of gonadotropins from the pituitary gland [101]. The role of kisspeptin in regulating reproductive maturation has also been described in non-mammalian vertebrates [102, 103], while a kisspeptin-type precursor was recently discovered in the cephalochordate *B. floridae* [10].

Analysis of the *A. rubens* neural transcriptome identified a 149-residue precursor protein comprising two putative kisspeptin-type peptides (ArKP1-2; Figure 1) [60]. ArKP1 shares a C-terminal NxxSxxLxF-NH_2_ motif with human kisspeptin. However, unlike human kisspeptin, ArKP1 has two cysteine residues in its N-terminal region that may form a disulphide bridge—this feature of ArKP1 also occurs in a putative kisspeptin-type peptide in the sea urchin *S. purpuratus*, and therefore, it may be a characteristic of echinoderm kisspeptins [60]. ArKP2 is similar to ArKP1 but it lacks the N-terminal pair of cysteine residues present in ArKP1 and has additional residues in the C-terminal region of the putative neuropeptide.

The discovery of the *A. rubens* kisspeptin-type precursor is consistent with the occurrence of kisspeptin-type receptors in non-chordates [9, 10], although both kisspeptin-type precursors and receptors appear to have been lost in urochordates and ecdysozoans [10]. The discovery of ArKP1 and ArKP2 provides an exciting opportunity to investigate the physiological roles of kisspeptins in an invertebrate for the first time.

### The first MCH-type neuropeptide to be discovered in a non-chordate

MCH was first discovered in teleost fish on account of its effect of inducing a change in body colour [104, 105]. MCH-type peptides have subsequently been identified throughout the vertebrates [106–108] and have been implicated in a range of physiological roles including the regulation of feeding, sleep and reproduction [109, 110].

Analysis of the *A. rubens* neural transcriptome identified an 88-residue precursor protein with a predicted 28-residue MCH-type peptide (ArMCH; Figure 1) [60]. The location of the putative MCH-type peptide in the C-terminal region of the precursor is likewise a characteristic of MCH-type precursors in vertebrates [111]. Furthermore, vertebrate MCH-type peptides have a conserved pair of cysteine residues that form a disulphide bridge and, accordingly, the presence of two cysteine residues in ArMCH indicates that the starfish peptide also has a disulphide bridge [112]. Identification of the *A. rubens* MCH-type precursor also facilitated identification of MCH-type precursors in the sea urchin *S. purpuratus* and the hemichordate *S. kowalevskii* [60].

The discovery of the *A. rubens* MCH-type precursor is consistent with the occurrence of MCH-type receptors in non-chordates including the cephalochordate *B. floridae* and the hemichordate *S. kowalevskii* [9, 10]. However, to date, MCH-type precursors and receptors have not been found in protostomes, which indicates that MCH-type neuropeptide signalling may be restricted to the deuterostomian branch of the animal kingdom [9, 10]. Thus, the discovery of a putative MCH-type peptide in *A. rubens* provides a unique opportunity to investigate the physiological roles of a MCH-type peptide in an invertebrate for the first time.

### Starfish reveal the evolutionary origins of paralogous GnRH and corazonin signalling pathways

GnRH is widely known as a regulator of reproductive maturation in the vertebrates [34, 35]. It has also been discovered that homologues of GnRH occur in invertebrates. These include AKH, red pigment concentrating hormone [31–33], corazonin (CRZ) [33, 113] and AKH/CRZ-related peptide (ACP), which are found in insects and other arthropods [114, 115]. The AKHs are a family of lipid-mobilizing hormones released during flight and locomotion in insects [31]. CRZ was discovered on account of its stimulatory effect on heart rate in cockroaches [116] but has been implicated in a range of functions in the arthropods (e.g. initiating ecdysis in moths via the release of pre-ecdysis-triggering hormone and ecdysis-triggering hormone) [117]. ACP is a paralogue of AKH that arose in a common ancestor of the arthropods. However, despite insights into its evolutionary origins, the function of ACP remains unclear [118]. Recently, there has been debate as to the relationship of CRZ to AKH, ACP and GnRH. For example, it has been proposed that AKH/ACP and CRZ neuropeptides are both orthologous to vertebrate GnRH [9, 29, 30]. However, other studies have been inconclusive in establishing this relationship [10, 115].

A GnRH-like peptide (pQILCARAFTYTHTW-NH_2_) that activates one of two CRZ-type receptors has been identified in the cephalochordate *B. floridae* based on analysis of genomic sequence data [119]. However, insect AKH also activates the same *B. floridae* CRZ-type receptor [120], and therefore, it was unclear whether there are distinct GnRH-type and CRZ-type neuropeptide signalling systems in deuterostomes.

The identification of precursors of two GnRH-like peptides in *A. rubens* [60] has provided new insights into this issue because it has been found that one of the peptides (pQIHYKNPGWGPG-NH_2_; structure confirmed by mass spectrometry) activates an *A. rubens* GnRH-type receptor and the other peptide (HNTFTMGGQNRWKAG-NH_2_; structure confirmed by mass spectrometry) activates an *A. rubens* CRZ-type receptor (Figure 1) [62]. Importantly, no cross-activation between the two ligand-receptor pairs was observed, demonstrating the existence of two distinct signalling systems [62]. These findings indicate that the evolutionary origin of the paralogous GnRH-type and CRZ-type signalling systems can be traced back to gene duplication in a common ancestor of the Bilateria.

## Conclusions and directions for future research

Genome-wide studies have begun to unravel the evolutionarily ancient origins of neuropeptide signalling systems [9, 10], and analysis of neuropeptide systems in echinoderms has provided some key insights. Thus, identification of ligand–receptor pairs in the sea urchin *S. purpuratus* and the starfish *A. rubens* has revealed how ancient gene duplications gave rise to the bilaterian NG peptide [82] and GnRH/CRZ [62] neuropeptide families, respectively. Looking ahead, echinoderm genome/transcriptome sequence data present us with many more interesting questions. For example, the presence of neuropeptide Y (NPY) and galanin-type receptors in the sea urchin genome indicates the presence of NPY and galanin-type peptides, but these have yet to be identified [9, 10]. Addressing these issues may be aided by analysis of sequence data from other echinoderms, including brittle stars (Class Ophiuroidea) and sea lilies/feather stars (Class Crinoidea) [98].

In conclusion, the availability of sequence data has provided a molecular phylogenetic framework to probe how orthologous neuropeptide systems are used to regulate physiological and behavioural processes in evolutionarily distant phyla. Looking forward into an era of post-genomic functional analysis of neuropeptide signalling, we anticipate that by virtue of their phylogenetic position as non-chordate deuterostomes, echinoderms will continue to provide us with many more missing pieces in the ‘jigsaw puzzle’ of neuropeptide evolution. Furthermore, with the unique perspective of a decentralized and pentaradial bauplan [47, 48], we expect some surprises!

Key Points

- Neuropeptides are evolutionarily ancient mediators of neuronal signalling controlling a range of physiological processes and behaviours.
- Genomic/transcriptomic analysis of neuropeptide signalling systems in echinoderms has recently provided key insights into neuropeptide evolution.
- Sequencing of the sea urchin *Strongylocentrotus purpuratus* genome enabled discovery of the first invertebrate TRH-type precursor, the first deuterostomian pedal peptide/orcokinin-type precursors and the unification of a bilaterian NG peptide family.
- Sequencing of the starfish *Asterias rubens* neural transcriptome enabled identification of 40 novel neuropeptide precursors, including the first kisspeptin and MCH-type precursors to be discovered outside of the chordates and the discovery of the first CRZ-type neuropeptide receptor to be deorphanized in a deuterostome.
- Discovery of neuropeptide signalling systems in echinoderms provides opportunities to investigate neuropeptide function in the unique context of a decentralized and pentaradial bauplan.

## Funding

This work was supported by Leverhulme Trust grant RGP-2013-351 and BBSRC grant BB/M001644/1 (awarded to M.R.E.).
